# Multistage Tool Path Optimisation of Single-Point Incremental Forming Process

**DOI:** 10.3390/ma14226794

**Published:** 2021-11-11

**Authors:** Zhou Yan, Hany Hassanin, Mahmoud Ahmed El-Sayed, Hossam Mohamed Eldessouky, JRP Djuansjah, Naser A. Alsaleh, Khamis Essa, Mahmoud Ahmadein

**Affiliations:** 1 School of Engineering, University of Birmingham, Birmingham B152TT, UK; Zhou.Yan@bham.ac.uk (Z.Y.); k.e.a.essa@bham.ac.uk (K.E.); 2 School of Engineering, Technology, and Design, Canterbury Christ Church University, Canterbury CT1 1QU, UK; 3 Department of Industrial Engineering, Arab Academy for Science Technology and Maritime, Alexandria 21599, Egypt; m_elsayed@aast.edu (M.A.E.-S.); hossam.eldessouky@aast.edu (H.M.E.); 4 College of Engineering, Imam Mohammad Ibn Saud Islamic University, Riyadh 11564, Saudi Arabia; jrdjuansjah@imamu.edu.sa (J.R.P.D.); naalsaleh@imamu.edu.sa (N.A.A.); maahmadein@imamu.edu.sa (M.A.); 5 Department of Production Engineering and Mechanical Design, Tanta University, Tanta 31512, Egypt

**Keywords:** SPIF, sheet metal, forming, incremental, FEA, tool path, optimisation

## Abstract

Single-point incremental forming (SPIF) is a flexible technology that can form a wide range of sheet metal products without the need for using punch and die sets. As a relatively cheap and die-less process, this technology is preferable for small and medium customised production. However, the SPIF technology has drawbacks, such as the geometrical inaccuracy and the thickness uniformity of the shaped part. This research aims to optimise the formed part geometric accuracy and reduce the processing time of a two-stage forming strategy of SPIF. Finite element analysis (FEA) was initially used and validated using experimental literature data. Furthermore, the design of experiments (DoE) statistical approach was used to optimise the proposed two-stage SPIF technique. The mass scaling technique was applied during the finite element analysis to minimise the computational time. The results showed that the step size during forming stage two significantly affected the geometrical accuracy of the part, whereas the forming depth during stage one was insignificant to the part quality. It was also revealed that the geometrical improvement had taken place along the base and the wall regions. However, the areas near the clamp system showed minor improvements. The optimised two-stage strategy successfully decreased both the geometrical inaccuracy and processing time. After optimisation, the average values of the geometrical deviation and forming time were reduced by 25% and 55.56%, respectively.

## 1. Introduction

Conventional sheet metal-forming (SMF) techniques such as drawing, stamping, rolling, and stretch forming are well-established mass production processes for a wide range of sheet metal products, especially in the automotive and aerospace industries. The main disadvantage of conventional SMF is the need to design and manufacture special dies with the required geometry of the product. The cost of the SMF dies is directly proportional to the complexity of the geometry [1]. Therefore, these techniques are not compatible with the customisation or personalisation of products. On the other hand, additive manufacturing (AM) techniques offer the possibility of customising products with complex geometries, allowing them to find applications in several sectors [2,3,4,5,6]. However, additive manufacturing for automotive and aerospace industries is incapable of manufacturing large and curved sheet metals, such as vehicle body panels. Other challenges restricting AM products in automotive and aerospace industries are the poor surface finish, limited part size and disparities in the production quality [7,8].

The demand for low/medium mass customisation is continuously growing as a promising approach for fabricating products with a high degree of customisation at a low production cost. However, the current capabilities of additive manufacturing and conventional sheet metal-forming are incapable of satisfying the needs for mass customisation [9]. The SPIF process can reduce production costs by eliminating the need for die manufacturing. It also offers a high degree of flexibility in customisation, especially for medium-sized production, resulting in die-less metal forming [10]. Single-point incremental forming is based on the conventional sheet metal spinning commonly used for the production of axisymmetric shape complex parts, without the need for using dies [11,12,13]. In SPIF, the metal sheet is plastically deformed into the desired shape through an incremental localised progressive manner by using a spherical head tool. The concept was first introduced by Leszak et al. [14], and the first trials were carried out in Matsubara labs, Japan. However, the process was advanced further and is currently used in several industries, such as aerospace [15], automotive [16] and marine [17]. The drawbacks of SPIF are around the fabricated parts’ poor dimensional and geometrical precision due to the absence of dies in the process, making it challenging to ensure tight dimensional tolerance. Other geometrical precision issues typically arise from sheet thinning and elastic spring back [18].

Recently, significant advancements have been achieved in the equipment front, such as using individual or in sync robots to improve the flexibility and precision of the process. Several researchers have studied the SPIF experimentally, analytically and/or numerically. The selection of the process parameters was shown to significantly affect the thickness distribution of the metal sheet. Duflou and colleagues [19] had experimentally investigated the effect of different parameters such as tool diameter, sheet thickness, part wall angle and vertical step size on the forming force in the SPIF process. In their study, they had used a simple truncated cone part and contour forming strategy. Their results demonstrated a solid relationship between the induced forming forces and the selected process parameters. Bambach et al. and Dejardin et al. reported the significance of four SPIF process parameters which define the formability of the metal sheet. These were sheet thickness, tool speed, tool diameter and forming strategy [20,21]. Different forming strategies lead to different strain distribution and, hence, variation in the formed part thickness. The tool path is the route through which the tool travels in order to deform the metal sheet to the required shape. Therefore, both the formed component geometry and the process tolerance strongly depend on the tool path. Contour and spiral tool path strategies are two forming strategies that are typically applied in the SPIF process [22]. Arfa et al. [23] compared the two tool path strategies, and they stated several advantages associated with adopting the spiral tool path strategy. This includes uniform formed part thickness, homogeneous strain distribution and reduced defects on the surface of the formed part. Other researchers had also reported the remarkable influence of the forming strategy on the quality of the formed part. Therefore, optimising the SPIF tool path has become an interesting research area [18,20,22].

Generally, most of the research investigating the tool path optimisation of the SPIF process focused on improving the forming strategy. The optimisation of the SPIF tool path initially concentrated on minimising the spring-back phenomenon and cutting down the tool trajectory. Although the experimental approach usually offers better accuracy, it is also much more time- and money-consuming. Essa [18] studied the use of a backing plate, tool path modification and a kinematic supporting tool to improve geometrical accuracy. Azaouzi and Lebaal [22] examined the spiral tool path strategy by optimising the weighting factor and the tool vertical tour number with the objective of controlling the tool path. They used the statistical response surface technique to optimise the spiral tool path. Through this optimisation, they successfully reduced the tool path length while improving the uniformity of the part thickness. Reese and Ruszkiewicz [24] realised that the spring-back of an aluminium truncated cone could be reduced by increasing the incremental step size in the z-direction. Recently, Maaß et al. [25] studied the influence of the step-down size on the characteristics of the forming mechanisms via numerical simulation. Their results indicated that increasing the step-down size reduced the part thickness and the geometrical accuracy. However, it was also shown to increase the waviness of the formed part [22,25]. Due to the dynamic and incremental concept of SPIF, it was reported that the process simulation often consumes considerable CPU time. Maidagan et al. and [26] Meier et al. [27] introduced the double-side incremental forming (DSIF) to improve the geometrical accuracy of the formed sheet. In this technique, two forming tools simultaneously work on the two sides of the sheet metal. The DSIF was found effective at improving geometric accuracy [28].

Gonzalez et al. [29] investigated the multistage incremental sheet forming to improve the formability and accuracy of SPIF of conical geometry. The results showed that multistage forming enhanced the geometric accuracy in the unformed areas. However, the sheet thickness was deteriorated compared to those formed using single-stage forming. Suresh et al. [30] managed to implement multistage incremental sheet forming experimentally to produce geometries with steep walls that were difficult to achieve using SPIF. Recent research introduced multistage forming as a technique to improve geometrical accuracy, spring-back and thickness distribution [29,31]. However, literature on optimising this technique statistically is lacking. The research question of this paper is related to how effective is the use of the design of experiments to optimise a multistage single incremental forming and what are the effects and interactions of multistage forming parameters on the geometrical accuracy and forming time?

## 2. Methodology

Finite element analysis was initially used and validated using experimental literature data. Step-down tool size and the forming depth have been varied in order to provide some insight. Initially, an FEA model of SPIF was built using an explicit solver and validated with experimental data in the literature. The part shape was a truncated aluminium alloy cone with a 180 mm diameter, 40 mm depth and 50° wall angle. The experiments were modelled using an ABAQUS/Explicit solver. Finally, the simulation results, such as the average geometrical deviation and the forming time in various simulation scenarios, have been used to analyse variance and identify the optimum parameters utilising the design of experiments (DoE) method and analysis of variance. The results obtained using the optimised multistage tool path parameters were compared to a single-stage one introduced by Maaß et al. [25].

### 2.1. FEA Modelling of SPIF Process

**Figure 1:**
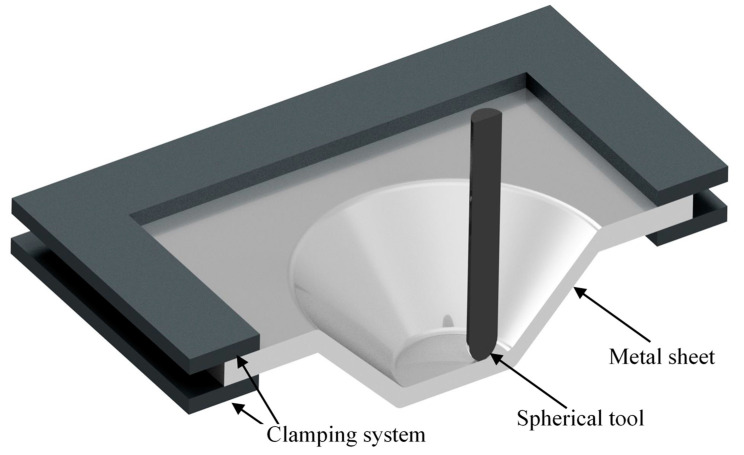
A schematic diagram of the SPIF system.

Finite element modelling (FEM) has been a powerful process modelling tool allowing the study of the deformation mechanism of the SPIF process and by implication to avoid restrictions associated with facilities and location. The sheet metal part is typically modelled using shell or solid elements. Solid elements are preferred for meshing the thickness direction to improve the accuracy of the results. However, this increased the computational time [21]. Cocchetti et al. [32] recommended the use of shell elements to save computational time. Explicit and implicit FEM solver techniques were investigated in the literature, and it was found that the explicit solvers were time-efficient, whereas the implicit method was accurate. Researchers have explored the use of implicit and explicit solvers aiming to reduce computational time. A simultaneous solution of equations in each time increment was used to solve the problem in the implicit solver. In the explicit FEA technique, the solution of the preceding step was employed to solve the succeeding increment. As a result, the implicit technique was reported to be more precise than the explicit solver due to eliminating error accumulation [23]. Therefore, using the explicit method primarily to explore the optimal range of process parameters, followed by employing the implicit solvers, could permit the prediction of more accurate results in a reasonable time. A schematic diagram of SPIF is illustrated in Figure 1. The spherical tip tool makes a sequence of contours and forms the desired shape incrementally into the metal sheet.

**Figure 2:**
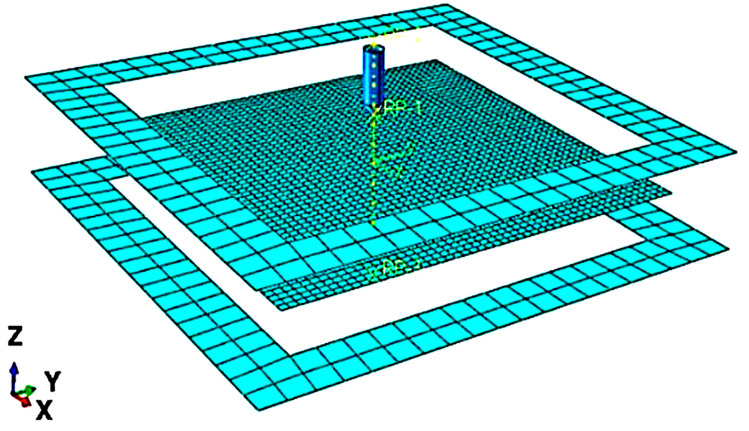
The simulation model of the SPIF process.

An asymmetric geometry represented the truncated cone, with a diameter of 180 mm, a depth of 40 mm and a wall angle of 50°, and it was constructed to simulate the SPIF in ABAQUS software. The sheet metal was defined as a (200 × 200 mm) blank sheet of 1.2 mm in thickness. The blank sheet was held between two blank holders and deformed using a spherical tool of 10 mm in diameter. Figure 2 shows the FEA model in which the metal sheet is considered a deformable body, whereas rigid bodies were assigned to the tool and the two clamping holders. The sheet material was aluminium 3003-O, with the following physical and mechanical properties: density ρ = 2700 kg/m^3^, Poisson’s ratio ν = 0.33, Young’s modulus E = 70 GPa, ultimate strength $\sigma_{u}$ = 95–135 MPa and yield stress $\sigma_{y}$ = 35 MPa [19]. The flow stress equation of the material can be calculated using the Swift-type hardening law, as follows:

(1) $$\bar{\sigma }=k{\left(\varepsilon_{0}+\bar{\varepsilon }\right)}^{n}$$

where k is the hardening coefficient, $\bar{\varepsilon }\;$is the effective accumulated plastic strain and *n* is the strain hardening exponent [23]. For aluminium 3003-O: k= 184 MPa, $\varepsilon_{0}$ = 0.00196 and n=0.224.

The velocity of the tool feed was maintained at 2000 mm/min to eliminate the effect of the tool speed on the process. It is typical to use a lubricant covering the metal sheet in the SPIF process, and this has been set as a friction coefficient of 0.09 between the forming tool and the metal sheet. In addition, the friction coefficient between the clamp, sheet and backing was set at 0.015. The tool path was initially defined by the movement in x, y and z directions. At each time increment, the coordinate of the tool reference point was initially generated using MATLAB and converted into displacement. The tool step-down was identified as 0.5 mm, and the explicit time integration was set as a nonlinear analysis in which the sheet metal was continuously loaded. In order to stabilise the time discretisation, the time increment was set as small as possible [32]. The maximum time increment was calculated by the smallest mesh size and the sound speed, as in Equation (2) [33]:

(2) $$\Delta t\approx \;\frac{L_{min}}{S}$$

where Δt is maximum stable time increment, S is the sound speed $\mathrm{and}\;L_{min}$ is the smallest mesh size. For any isotropic shell element, S can be calculated using:

(3) $$S=\sqrt{\frac{E}{\left(1-\nu^{2}\right)\rho }}$$

where ρ is the material density (ton/mm^3^), ν is the Poisson’s ratio and E is the Young’s modulus (MPa). As shown from the above equation, as the density increases, the computational time decreases. However, further increases in mass scaling can result in inaccurate results. Therefore, the mass scaling factor was adapted according to the minimum mesh size in order to stabilise the time integration and save computational time. This resulted in adjusting the step time increment to around 10^−5^, and the use of the shell element as the mass scaling was found suitable. The critical time step was controlled by the size of the in-plane elements rather than the thickness of the sheet metal [32]. After several simulation convergence trials, the simulation results were comparable to the results by Duflou et al. [19] and Arfa et al. when the mesh size of the part was 4 mm, the mesh size of the clamp and backing were 12 mm and the mass scaling was 4 × 10^4^ [23]. Moreover, the kinetic energy was less than 5% of the model internal energy, indicating a negligible effect of the mass scaling on the results’ accuracy. Therefore, a conservation analysis was carried out to reduce the computational time while not compromising the model accuracy. It was suggested that the suitable mesh size for the part would be 4 mm, with a mass scaling of 4 × 10^4^ and 2500 elements.

### 2.2. Model Validation

The model’s reliability was assessed by comparing the current modelling results of a single-stage model with the experimental and simulation data in the literature [19].

The wall thickness of the part can be calculated using the sine principle, as shown in Equation (4) [23]:

(4) $$T_{part}=T_{0}\sin\left(90-\alpha \right)$$

where $T_{part}$ is the formed part thickness, $T_{0}$ is the thickness of the metal sheet and α is the cone angle.

**Figure 3:**
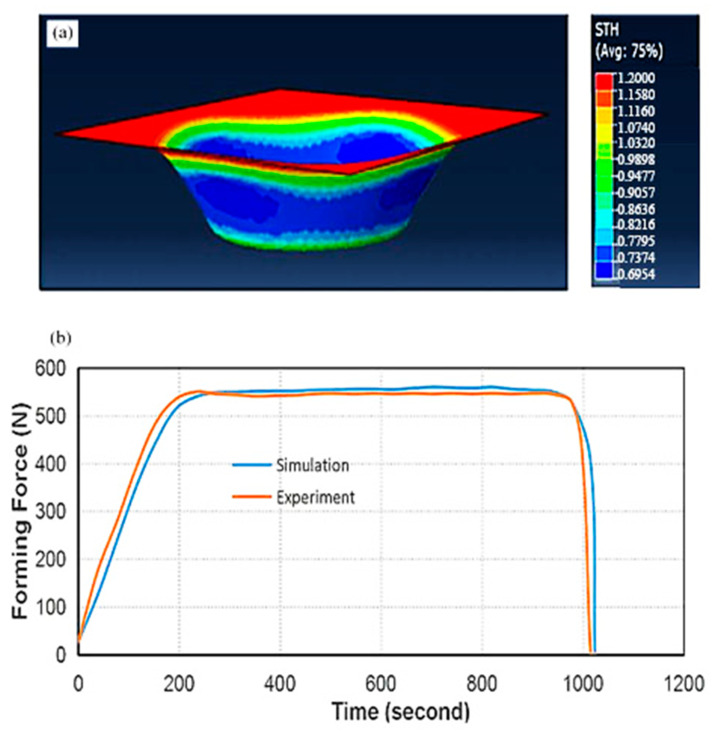
(**a**) The formed part thickness distribution, and (**b**) the forming force plot from the present simulation and the experimental results from the literature. The experimental graph was reused with permission from [19]. Elsevier.

The estimated thickness of the formed part was found to be ≈0.77 mm. The range of the formed part thickness, obtained from the present simulation and shown in Figure 3a, was from 0.6954 to 1.200 mm, which was comparable to that reported in the literature (0.7216–1.200 mm) [23].

Duflou et al. [19] used single-point incremental forming forces using a table-type dynamometer. To ensure an accurate comparison, the current study used the same setup parameters as those introduced by Duflou et al. [19], such as sheet material Al 3003-O, the thickness of 1.2 mm, a cone of 180 mm in diameter, 40 mm depth, 10 mm tool diameter and 50° wall angle. They optimised four different process parameters and their effect on the forming forces: the tool diameter, the sheet metal thickness, the vertical step size and the parts’ wall angle. The approximate mean thickness of the formed part in the current study and in the literature [19], represented by the blue areas in Figure 3a, were 0.7374 and 0.7615 mm, respectively. Both values are considered accurate compared to the theoretical value (0.77 mm) with an error of 1.4% and 5.5%, respectively.

Figure 3b compares the forming force versus time in the current study with corresponding values found in the literature [19]. As shown, the two curves have a similar trend and coincide at the same maximum value. Therefore, the current simulation can be considered a valid model and can be reliably employed to investigate the multistage tool path optimisation.

## 3. Optimisation of the Multistage SPIF Process

### 3.1. Multistage Parameters

**Figure 4:**
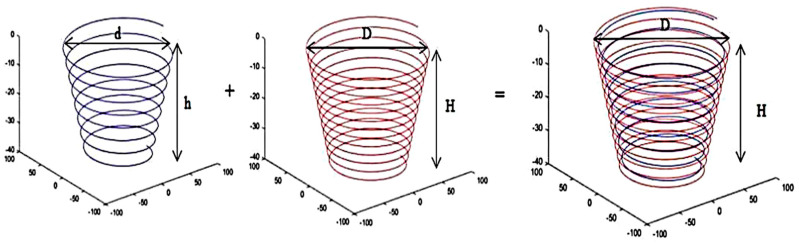
The two forming stages of the proposed SPIF process.

Two spiral tool paths were considered in this study (one for each forming strategy). The paths’ parameters are the depths (*h*, *H*) and the maximum diameters (*d*, *D*). Lowercase and capital letters are associated with forming strategies 1 and 2 respectively, see Figure 4. The maximum depth and diameter during the second stage (*D*, *H*) must be the same as the part dimensions, which are 40 and 180 mm, respectively. In addition, the part should have the same wall angle as the desired geometry after the first stage to ensure high geometrical accuracy. Earlier studies had explored the effect of the step size in the range of 1.875 to 5.625 mm and found that a step size of 5 mm was optimal [22,25]. Generally, increasing the step-down size reduces the geometrical accuracy and the forming time. The step-down size of forming stages 1 and 2 (denoted as ∆*z*1 and ∆*z*2) have been considered in a range from 1 to 5 mm. Accordingly, the range of *d* and the value of *h* were calculated through Equations (5) and (6) and were found to be 35–39 and 178 mm, respectively.

(5) H−h ≤Δz

(6) $$d\leq D-\frac{2\Delta z}{\tan50}$$

The spiral tool path parametric equation can be written as follow:

(7) $$\{\begin{array}{c} X\left(\beta \right)=R\left(\beta \right)\cos\beta \;\\ Y\left(\beta \right)=R\left(\beta \right)\sin\beta \;\\ Z\left(\beta \right)=\frac{Z_{max}}{2\pi } \end{array}$$

where R(β) is the radius as a function of the spiral angle $\beta ,\;n\;\mathrm{is}\;\mathrm{the}\;\mathrm{vertical}\;\mathrm{increment}=\frac{Z_{max}}{\Delta z},\;0≦\beta ≦2\pi n\;\mathrm{and}\;Z_{max}$ is the maximum forming depth, which is calculated using Equation (8):

(8) $$R\left(\beta \right)=r+\frac{Z\left(\beta \right)}{\tan\left(\theta \right)}$$

where *r* is the spherical tool radius and *θ* is the wall angle. Three parameters have been considered. Those are the vertical step-down sizes during the first and the second forming stages (∆*z*1 and ∆*z*2, respectively), and the depth of the first forming stage (h), in which the ranges are 1≦Δz1 and Δz2≦5, and 35≦h≦39.

Maaß et al. [25] investigated the relationship between the tool path parameters such as the vertical step size, ∆*z*, and the part geometry of a similar truncated cone. The authors found that the optimum step size ratio for a single-stage incrementing forming ∆*z*/tool radius is 0.25 for an accurate part geometry. Therefore, an initial value for ∆*z* of 1.25 mm was employed in the current study, in comparison to the multistage optimised values.

### 3.2. The Response Surface Method (RSM)

The design of experiments is a statistical approach for designing and optimising processes with multiple input parameters, and it has been used extensively in sheet metal forming as well [34,35]. The response surface method was used to statistically investigate the effect of the process parameters using the two forming stages’ spiral tool paths and to find the optimised parameters, as shown in Figure 4. The aim was to deform the sheet metal evenly and reduce spring-back with a minimum forming time. To achieve this aim, the Box–Behnken DoE was used to create an experimental plan with minimum trials. The Box–Behnken method is a statistical design used for the response surface approach to ensure that each parameter is placed at one of three equally spaced levels, such as −1, 0 and +1 [36].

A second-order regression equation can express the response surface function “Y”. The order of the regression equation is typically kept as low as possible. Typically, the accuracy of second-order regression is the lowest accurate order, see Equation (9):

(9) $$Y=b_{0}+\sum b_{i}x_{i}+\sum b_{ii}x_{i}^{2}+\sum b_{ij}x_{i}x_{j}\;\;\;$$

where the factors x_i_ are the process parameters; on the other hand, *b_0_, b_i_, b_ii_* and *b_ij_* are the regression equation coefficients determined using the least-square technique. Design-Expert V 7.0.0 (Stat-Ease Inc., Minneapolis, MN, USA) was applied to implement the DoE approach.

Three parameters were deemed appropriate in the study: vertical tool increment of the first forming stage, vertical tool increment of the second forming stage and the depth of the first forming stage. According to the Box–Behnken design, three levels of each parameter were considered, see Table 1. As shown, the 3 levels were 0 as the middle level, 1 as the high level and −1 as the lower level [32]. Furthermore, three centre points were considered (to permit the determination of the experimental error). This resulted in 15 parametric combinations, see Table 2. In this study, two responses: the average geometric deviation of the formed part and the forming time, were optimised, knowing that the geometric deviation of the part was calculated using the average distance between the formed and designed shapes [23,24].

## 4. Results

### 4.1. Analysis of Geometric Deviation

**Figure 5:**
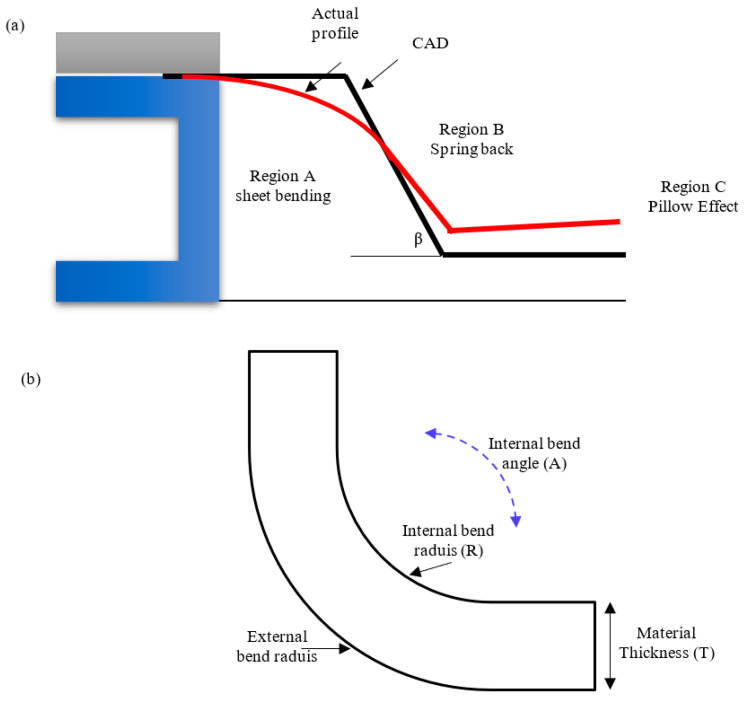
(**a**) A schematic diagram of the actual and ideal geometries. (**b**) Sheet metal parameters.

Three distinct geometric deviations were found at three regions, see Figure 5a. The deviation in region A was created by bending the metal sheet, while the ones found in region B were due to the spring-back effect. Finally, the pillow effect deviation at the base of the part (region C) was a result of the change in the transverse stress/strain [32,36]. The sheet metal parameters such as the internal bend angle (*A*), internal bend radius (*R*) and the sheet thickness (*T*) are shown in Figure 5b.

**Figure 6:**
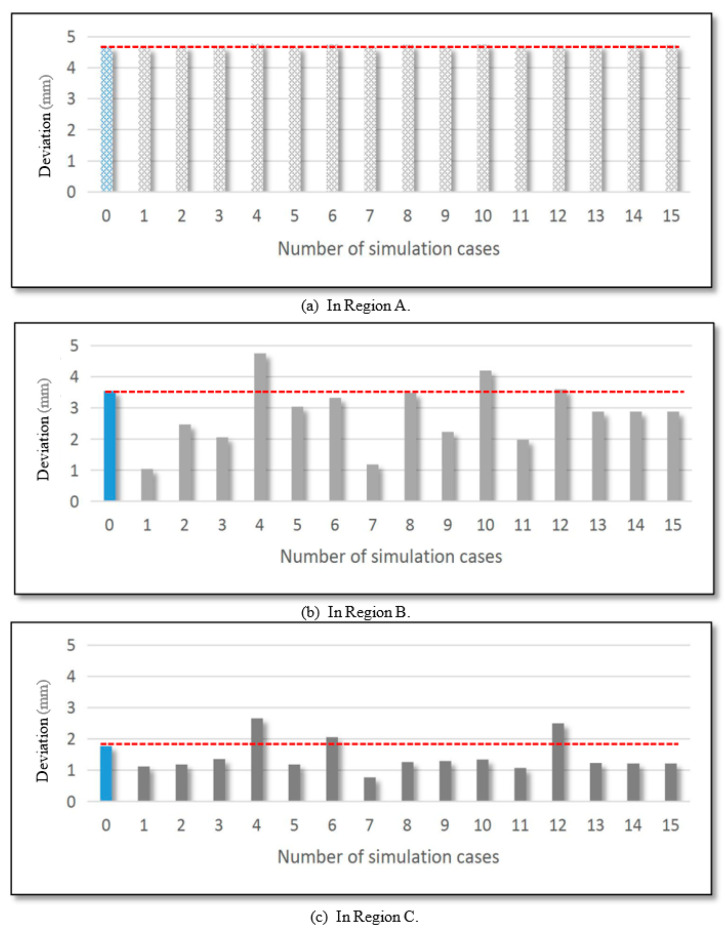
The maximum geometric deviation in region A, region B and region C.

To assess the part quality in the fifteen simulation trials, the maximum error in each of the three regions was measured, see Figure 6. It was found that the maximum geometrical deviation in region A was about 4.70 mm for all simulation scenarios, which did not improve compared to the single stage (4.67 mm). There was a significant variation in the error values in region B, which ranged from 1.04 to 4.75 mm. The maximum geometrical deviation in region C varied from 0.7688 to 2.6546 mm. In conclusion, the second forming stage improved the geometric accuracy in regions B and C while not deteriorating in region A.

### 4.2. Analysis of Variance (ANOVA)

Results of the average deviation and forming time are listed in Table 2. The least-square fitting *R*2 was employed to define the model fit [37]. According to the Box–Behnken design, the average geometrical deviation and forming time fit quadratic models with the least-square fitting *R*2 of 97% and 95%, respectively. This suggests that the models accurately describe the relationship between the input parameters and the outputs. The two models are functions of the vertical step-down sizes during the first forming stage (∆*z*1), the second forming stage (∆*z*2) and the depth during the first forming stage (*h*), see Equation (10).

(10) $$Response=b_{0}+b_{1}\left(\Delta z1\right)+b_{2}\left(\Delta z2\right)+b_{3}\left(h\right)+b_{4}\left(\Delta z1\Delta z2\right)+b_{5}\left(\Delta z1h\right)\;+b_{6}\left(\Delta z2h\right)+b_{7}{\left(\Delta z1\right)}^{2}+b_{8}{\left(\Delta z2\right)}^{2}+b_{9}{\left(h\right)}^{2}$$

where *b_0_* is the average of the levels, and *b*_1_, *b*_2_*…, b*_9_ are the models’ coefficients. Least-squares fitting, an approach for best curve fitting by minimising the total squares of the errors, was used to analyse the equation data shown in Table 3 and define the coefficients. The coefficients of the surface response model for the two outputs are listed in Table 3.

The null hypothesis, which presumes that the process parameters have no effect, is rejected when the *p*-value is less than 0.05 (95% confidence level). As a result, parameters with *p*-values ≤ 0.05 are considered significant. The calculated *p*-values of all the parameters and interactions are listed in Table 4. The ANOVA results show that the most significant parameters are the vertical increments during stages one and two, the interaction between both increments and the depth of forming during stage one. Furthermore, the forming time was found to be significantly affected by the vertical increments of both forming stages.

**Figure 7:**
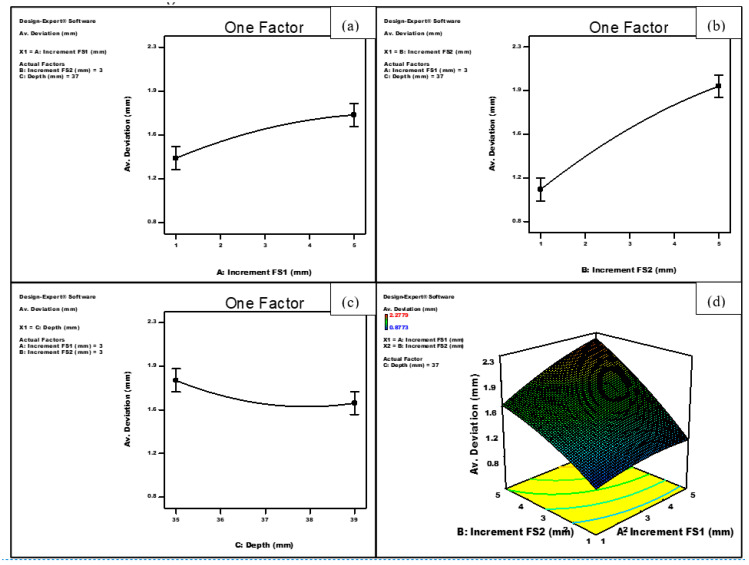
Effect of (**a**) increment of forming stage 1 (FS1), (**b**) increment of forming stage 2 (FS2), (**c**) depth of forming stage 1 and (**d**) the interaction between the increments of both forming stages on the average deviation.

**Figure 8:**
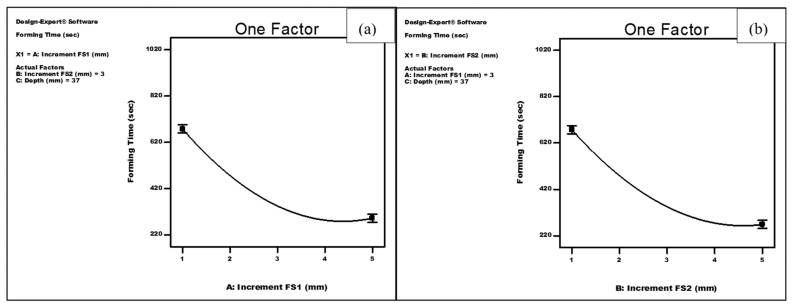
Effect of (**a**) increment of forming stage 1 (FS1) and (**b**) increment of forming stage 2 (FS2) on the forming time.

Figure 7a–c show the effect of the increment of the first forming stage (FS1), increment of the second forming stage (FS2) and the depth of the first forming stage on the average deviation using a quadratic model. It can be noted that the average deviation increased consistently with increasing any of the vertical increments of stages 1 and 2. In addition, the depth of the forming stage 1 was shown to have a slight adverse effect on the average deviation. Finally, the model shows that the interaction of the increments during the two forming stages was also significant. The geometric deviation significantly increases as the increment of any of the forming stages increases, Figure 7d. On the other hand, and as shown in Figure 8a,b, each of the increments of forming stages 1 and 2 were significant on the forming time, and the relationship between them follows a quadratic model. Both factors were shown to have virtually the same inverse effect on the forming time. At a depth of stage 1 of 37 mm, increasing the increment of forming stage 1 from 1 to 5 mm (at a constant value of the increment of forming stage 2 of 3 mm) resulted in a reduction of the forming time from 677 to 292 s, while the same increase of the increment of forming stage 2 (at a constant value of the increment of forming stage 1 of 3 mm) caused the forming time to decrease from 677 to 269 s.

### 4.3. Optimisation of Process Parameters

The process parameters of the two-stage process were optimised. The objective function is to minimise both the geometrical deviation and the forming time. However, two different priorities were used in the objective function. The geometrical accuracy was defined with high priority, while the forming time was defined with low priority. Therefore, if both objectives have an inverse relationship, a trade-off will be made. In this case, the geometrical accuracy objective function will be met at the expense of the forming time to obtain a valid solution.

**Figure 9:**
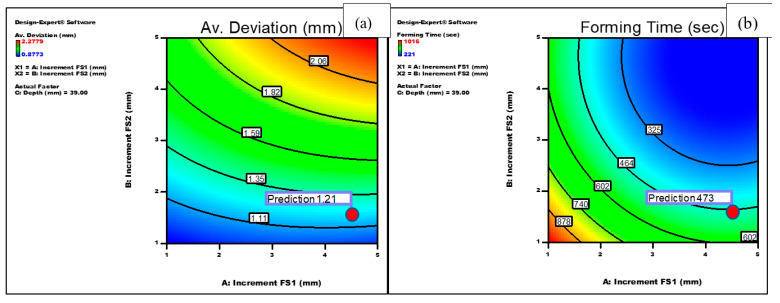
Predicted optimum process parameters for minimising the process responses (**a**) average deviation and (**b**) forming time.

The experimental data were analysed, and the genetic algorithm (GA) was employed to calculate the process parameters [38]. The genetic algorithm is a search optimisation technique that is inspired by natural evolution. The geometrical deviation and forming time shown in Equation (10) and the corresponding constants listed in Table 3 were simultaneously solved, and the contour plot of the optimisation is shown in Figure 9. The model shows that the optimum values of the vertical increment of stage 1, vertical increment of stage 2 and the depth of forming stage 1 would be 4.5, 1.59 and 39 mm, respectively. At these values, the predicted average deviation and forming time were 1.21 mm and 473 s, respectively.

### 4.4. Validation and Comparison

**Figure 10:**
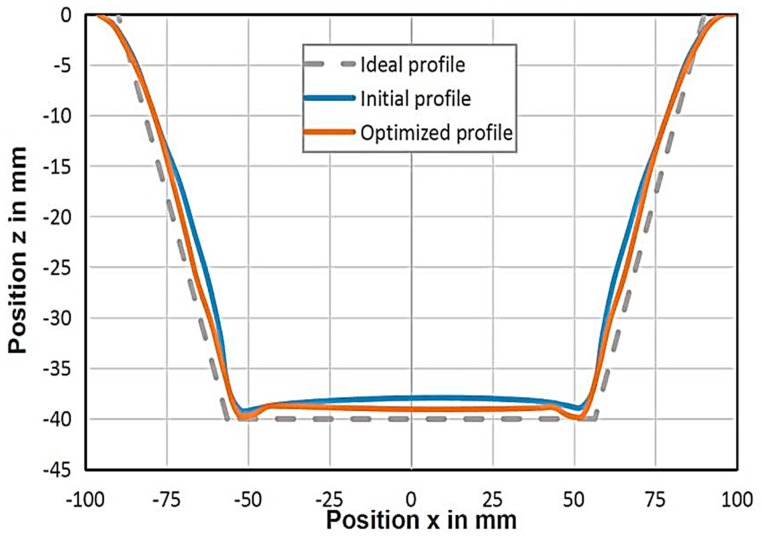
Section of the formed part produced using the single-stage process [25] and the optimised multistage process and ideal part.

**Figure 11:**
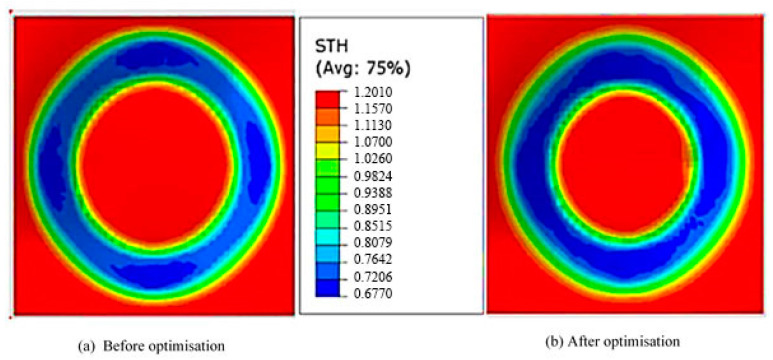
Thickness distribution of the (**a**) single-stage and (**b**) the optimised multistage SPIF process.

The aim of using the DoE optimisation was to minimise the geometrical deviation and forming time. The optimum process parameters were ∆*z*1 = 4.5 mm, ∆*z*2 = 1.6 mm and *h* = 39 mm. This set was employed in the FE model, and its effect on the part geometrical deviation and forming time is listed in Table 5. It was found that both the forming time and geometrical deviation were reduced by about 56% and 25%, respectively. Figure 10 shows the part geometry created by the optimised tool path parameters of a single-stage model created by Maaß et al. [25] and the current optimised process. The geometry of the formed part obtained using the current study’s optimised parameters was found more accurate to the ideal profile than the one obtained using the single-stage process [25], especially the regions along the part wall and the base. The deviations in the geometry caused by the spring-back effect and the transverse strain/stress in regions B and C were slightly penalised. However, the change in the geometrical deviation in region A, caused by sheet bending, was found negligible. Figure 11 demonstrates a comparison of the part thickness distribution between the single-stage and double-stage optimised processes. The minimal part thickness of the optimised formed part was decreased by 1.6%, resulting in a more uniform thickness compared with the single-stage process.

### 4.5. Forming Force

**Figure 12:**
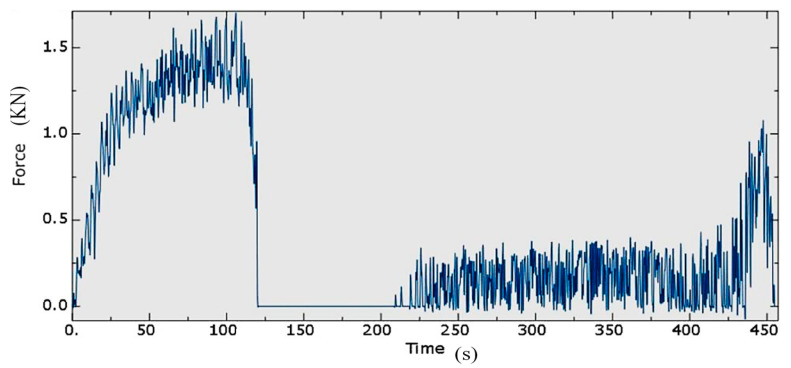
The forming force plot after optimisation.

The forming force calculated using the optimised SPIF process parameters, shown in Figure 12, increases during the first stage then breaks at ~120 s as the tool trajectory moves between the two forming stages. The figure also shows that the forming force during the first forming stage of the part is more significant than that of the second stage, as the deformation primarily occurs during the first stage. In the second stage, the tool deforms the sheet metal slightly compared to the first stage. Hence, the small reaction force in the second forming stage works better in improving the part geometry and the wall thickness. This is because, after the first stage, there is still an undeformed depth, in which the forming force increases. As the forming depth in the first stage increases, the peak of the forming force in the second stage decreases. In addition, the forming force in the first stage rises rapidly and then experiences almost a flat plateau, as shown in Figure 12. On the other hand, the forming force in the second stage initially increases, stabilises for a short time and then increases rapidly at the end of the second stage. When the step size of the second stage and the undeformed depth are small, the strain and stress near the part base become relatively small by the end of the second forming stage, making the pillow effect phenomenon negligible.

## 5. Discussion

The contact between the tool and the metal sheet occurs within region B, where the metal sheet has been forced into the ideal shape by a tool progressing according to the designed path. With the tool movement, a sheet metal part is continuously free from constraints, and the residual stress makes it deform without a forming force. Hence, the actual part deflects further from the ideal profile, and the build-up of the local spring-back increases the geometrical deviation. Typically, the increase in the forming force increases the residual stress. For parts created in a single stage, local deformation becomes more significant as the residual stresses increase. However, after introducing the second forming stage, the developed local spring-back is small, which reduces the geometric deviation in region B [20]. The forming force in the second stage is smaller than in the first stage, though it is still sufficient to deform the metal sheet into the desired profile. As the forming force and the associated residual stress in the second stage become small, the deformation also becomes small, and the part thickness deforms more uniformly than the first stage.

Although the tool and sheet metal do not directly connect in region *A*, the bending occurs due to deformation in region *B*. The length of the neutral line in region *A* can be denoted by the bend allowance, as shown in Equation (11):

(11) $$BA=A\left(\frac{\pi }{180}\right)\left(R+\left(KT\right)\right)$$

where R is the inside bend radius, A is the bend angle in degrees, T is the material thickness and K is the K-factor, which typically has a value between about 0.3 and 0.5. From Equation (11), it can be assumed that the bend allowance does not depend on the forming force. Therefore, the geometric deviation in region A is less dependent on the process parameters. On the other hand, the pillow effect that develops in region C develops from the bending of the metal sheet (due to in-plane stresses) [39]. This is because the material is largely deformed in the transverse direction within the tool vicinity. Consequently, it flows toward the metal sheet centre, which causes a significant pillow effect. The undeformed depth from the first stage can reduce the development of the pillow effect by restricting material plastic deformation near the base of the part.

Although the part dimensional accuracy is enhanced by using the proposed two-stage forming strategy, there is still a deviation between the formed part and the ideal shape, particularly near the area between the formed part and the clamping. As a result, the pillow effect becomes more evident as the size of the step-down increases. Further improvements in the design of the process can be considered in future work. For example, the tool path can be extended to the sheet centre point, reducing the pillow effect. In addition, the tool path start-points and endpoints of the two forming stages can be the same in order to reduce the spring-back effect due to the repeated forming. Furthermore, the distance between the forming and the clamped area can be reduced to minimise the sheet bending at the maximum diameter [40].

## 6. Conclusions

A two-stage forming strategy in SPIF was introduced and optimised to reduce the geometrical deviation and the processing time compared to those manufactured using a single forming tooling. A simulation model of the SPIF has been developed and solved using an explicit finite element analysis to study the optimal tool path for a truncated cone. The design of experiments using a response surface method was used to optimise the proposed two-stage forming strategy. The simulation results showed that the two-stage forming technique could significantly reduce both the geometrical deviation and the forming time. The step-down size in each forming stage was found to be the most significant parameter that affects the SPIF process’ formability. Meanwhile, the step size of the second stage affected the part accuracy more than the step size of the first stage. The proposed and optimised two-stage forming strategy can reduce the geometric deviation caused by the spring-back and pillow effect while having an insignificant effect on those caused by sheet bending near the part and the clamp. The forming time and part geometric deviation were reduced by 56% and 25%, respectively. In addition, the part thickness distribution was found more uniform after optimisation, and the minimal thickness decreased by 1.6%.

## Figures and Tables

**Table 1:** Factors and levels used in the DoE.

Factors	Process Parameters	−1	0	+1
∆*z*1 (mm)	Tool vertical increments of the first forming stage	1	3	5
∆*z*2 (mm)	Tool vertical increments of the second forming stage	1	3	5
*h (*mm*)*	Depth of the first forming stage	35	37	39

**Table 2:** Box–Behnken design parameters with calculated average deviation and forming time.

Run	∆*z*1	∆*z*2	*h*	Average Derivation	Forming Time
	mm	mm	mm	mm	(s)
1	5	3	39	1.6594	278
2	3	1	39	0.9792	658
3	3	5	35	2.077	258
4	3	3	37	1.5967	345
5	1	1	37	0.8773	1016
6	5	1	37	1.1333	633
7	3	3	37	1.5997	345
8	3	3	37	1.6056	345
9	5	5	37	2.2779	221
10	5	3	35	1.8586	258
11	1	3	39	1.4476	689
12	1	5	37	1.5239	580
13	1	3	35	1.5967	645
14	3	1	35	1.3892	645
15	3	5	39	2.0536	261

**Table 3:** Coefficients of the response surface models of the average deviation and forming time.

Coefficient	Average Deviation Model	Forming Time Model
*b*0	+40.94938	−4769.56250
*b*1	+0.21402	−254.87500
*b*2	−0.64276	−275.00000
*b*3	−2.06886	+330.50000
*b*4	+0.031125	+1.50000
*b*5	−0.00313125	−1.50000
*b*6	+0.024162	−0.62500
*b*7	−0.016468	+34.93750
*b*8	−0.020424	+31.93750
*b*9	+0.026445	−4.31250

**Table 4:** The *p*-values of the individual parameters and the interactions for the two outputs.

Model Parameter	*p*-Value	
	Average Deviation	Forming Time
∆*z*1	**0.0017**	**<0.0001**
∆*z*2	**<0.0001**	**<0.0001**
*h*	**0.0237**	0.1265
∆*z*1∆*z*2	**0.0340**	0.4722
∆*z*1*h*	0.7826	0.4722
∆*z*2*h*	0.0746	0.7592
*(*∆*z*1*)*^2^	0.2012	<0.0001
*(*∆*z*2*)*^2^	0.1276	<0.0001
*h* ^2^	0.0645	0.0846

Bold values indicate statistically significant process parameters (*p*-value < 0.05).

**Table 5:** Comparison between the forming time, average deviation and minimum sheet thickness.

Parameters	Single Stage Using ∆*z* = 1.25 mm, [25]	Optimised Solution	Reduction
Forming time (s)	1024	455	55.56%
Average deviation (mm)	1.5438	1.2351	25%
Minimum sheet thickness (mm)	0.6954	0.6770	1.6%

## Data Availability

The data underlying this article will be shared on reasonable request from the corresponding author.
